# Woman-centered research on access to safe abortion services and implications for behavioral change communication interventions: a cross-sectional study of women in Bihar and Jharkhand, India

**DOI:** 10.1186/1471-2458-12-175

**Published:** 2012-03-09

**Authors:** Sushanta K Banerjee, Kathryn L Andersen, Rebecca M Buchanan, Janardan Warvadekar

**Affiliations:** 1 Ipas India, E-63 Vasant Vihar, New Delhi, India; 2 Ipas, 300 Market St., Suite 200, Chapel Hill, NC 27516, USA; 3 Westat, 1600 Research Blvd., Rockville, MD 20850, USA

## Abstract

**Background:**

Unsafe abortion in India leads to significant morbidity and mortality. Abortion has been legal in India since 1971, and the availability of safe abortion services has increased. However, service availability has not led to a significant reduction in unsafe abortion. This study aimed to understand the gap between safe abortion availability and use of services in Bihar and Jharkhand, India by examining accessibility from the perspective of rural, Indian women.

**Methods:**

Two-stage stratified random sampling was used to identify and enroll 1411 married women of reproductive age in four rural districts in Bihar and Jharkhand, India. Data were collected on women's socio-demographic characteristics; exposure to mass media and other information sources; and abortion-related knowledge, perceptions and practices. Multiple linear regression models were used to explore the association between knowledge and perceptions about abortion.

**Results:**

Most women were poor, had never attended school, and had limited exposure to mass media. Instead, they relied on community health workers, family and friends for health information. Women who had knowledge about abortion, such as knowing an abortion method, were more likely to perceive that services are available (β = 0.079; p < 0.05) and have positive attitudes toward abortion (β = 0.070; p < 0.05). In addition, women who reported exposure to abortion messages were more likely to have favorable attitudes toward abortion (β = 0.182; p < 0.05).

**Conclusions:**

Behavior change communication (BCC) interventions, which address negative perceptions by improving community knowledge about abortion and support local availability of safe abortion services, are needed to increase enabling resources for women and improve potential access to services. Implementing BCC interventions is challenging in settings such as Bihar and Jharkhand where women may be difficult to reach directly, but interventions can target individuals in the community to transfer information to the women who need this information most. Interpersonal approaches that engage community leaders and influencers may also counteract negative social norms regarding abortion and associated stigma. Collaborative actions of government, NGOs and private partners should capitalize on this potential power of communities to reduce the impact of unsafe abortion on rural women.

## Background

Unsafe abortion, "a procedure for terminating an unwanted pregnancy either by persons lacking the necessary skills or in an environment lacking the minimal medical standards, or both" [1], is a neglected women's health issue in India and in many developing nations. Of the 6.4 million abortions performed in India in 2002 and 2003, 3.6 million (56%) were unsafe [2]. Worldwide, approximately 42 million pregnancies each year end in abortion [3], with 21.6 million of these abortions taking place under unsafe conditions [4]. Nearly all unsafe abortions (95-97%) occur in developing countries [4,5].

Unsafe abortion is associated with maternal mortality. In India, 12,000 deaths each year result from abortion-related complications [6]. Estimates for the contribution of unsafe abortions to maternal death in India vary from 9-20% [2,7-14]. Globally, it is estimated that unsafe abortions result in 47,000 deaths annually, and approximately 13% of all maternal deaths worldwide are attributable to unsafe abortion [4]. Leading causes of death include hemorrhage, infection, and poisoning from substances used to induce abortion [15].

Unsafe abortions are also strongly associated with maternal morbidity from complications such as hemorrhage, sepsis, peritonitis, and trauma to the cervix, vagina, uterus, and abdominal organs [15]. Morbidity from unsafe abortion is considered a serious problem in India [2,16]. Globally, high proportions of women (20-50%) who have unsafe abortions are hospitalized for complications [17]. Common long-term health problems caused by unsafe abortion include chronic pain, pelvic inflammatory disease, tubal blockage and secondary infertility [15]. Other potential consequences include an increased chance of ectopic pregnancy, spontaneous abortion, or premature delivery in subsequent pregnancies [15,18].

Almost all abortion-related deaths are preventable when performed by a qualified provider using correct techniques under sanitary conditions [5]. Recognizing the preventable nature of most maternal mortality and morbidity related to unsafe abortion [15,19,20], the Indian parliament passed the Medical Termination of Pregnancy (MTP) Act in 1971 [21]. This relatively liberal law permits a woman to seek an abortion to save her life, preserve her physical and mental health, for economic or social reasons, and in cases of rape or incest, fetal impairment, or when pregnancy results from contraceptive failure [12]. Subsequent amendments in 2002 and 2003 have aimed to expand safe services by devolving abortion service regulation to the district level, changing physical requirements for facilities providing first trimester abortions, and allowing medical abortion at facilities not approved for surgical abortion [21].

In addition to these legal and policy interventions, a number of interventions to increase the availability of safe abortion services have been implemented in India. For example, Ipas, a global non-profit reproductive health organization focused on safe abortion and women's reproductive rights, has helped establish 84 public sector and 5 private sector comprehensive abortion care training centers in India. More than 4209 providers, 2656 with a Bachelor of Medicine, Bachelor of Science (MBBS) degree and 1553 providers who are specialists in obstetrics and gynecology, have been trained, and three-fourths of providers trained by Ipas currently provide abortion services. Along with the Government of India (GoI), many non-governmental organizations (NGOs) like Janai, Pathfinder, Family Planning Association of India, and Parivar Seva Sanstha are also intervening to improve access to safe abortion services.

Unfortunately, these policy and service delivery interventions have not led to a significant reduction in unsafe abortion or related maternal mortality and morbidity in India [22], primarily because of limited access to and utilization of safe abortion services. While three-fourths of the Indian population live in rural areas, abortion services are rarely available at rural health facilities because trained doctors are not available to staff them [23,24]. Available safe abortion services are underutilized due to numerous individual and community-level factors, such as lack of awareness of the legality of abortion, limited understanding on the implications of unsafe abortion and lack of information on availability of safe providers and methods [25].

One way to address the gap between service availability and utilization is through behavior change communication (BCC) interventions. The theory behind BCC interventions is that by using communication channels to promote healthful behaviors and by creating a supportive environment, individuals will be able to act on these health-promoting behaviors [26]. A key goal of behavior change programs is to increase individuals' self-efficacy to engage in these health-promoting behaviors [27]. Though BCC interventions have successfully been used in India to increase knowledge of contraceptive use, immunization and HIV/AIDS [28,29], they have rarely been used to increase awareness of abortion issues. In rural Maharashtra, an intervention was developed to increase access to safe abortion services using both facility-based and community-based approaches [30]. This intervention included a BCC component, and though a formal impact evaluation was not conducted, evidence suggests that this type of community-based education campaign can be effective in increasing demand for abortion services in India [30].

The Behavioral Model of Health Services Use [31-33] is a useful conceptual model for understanding how interventions such as BCC campaigns can increase access to safe abortion services. Andersen delineates between potential access, which he defines as having enabling resources, and realized access, defined as use of health services [32]. The focus of this paper is on potential access since the goal of BCC interventions is to improve access to services by creating an enabling environment. Andersen argues that enabling resources are a measure of potential access because when they are present, the likelihood of service utilization increases [32]. Enabling resources include both community and personal resources such as trained health personnel and facilities nearby, knowledge of how to obtain the available services, financial resources to obtain services and social support for care-seeking [32]. BCC interventions aim to build these enabling resources by increasing knowledge and creating an environment in which women are supported in using available safe abortion services when they are needed.

In order to design effective interventions, public health professionals need to understand what enabling resources are in place from the perspective of women of reproductive age. This includes an understanding of the characteristics of women who use or seek access to existing services [34], their needs [34], and an understanding of the dynamics of their decision-making processes related to unwanted pregnancy and abortion [35]. The purpose of this study is to develop an evidence base for understanding accessibility of safe abortion services in Bihar and Jharkhand, India from the perspective of rural Indian women. This woman-centered perspective will be ascertained through the following research questions:

1. What are the socio-demographic, economic, and reproductive characteristics of women in four selected districts in Bihar and Jharkhand?

2. To what extent are women in Bihar and Jharkhand exposed to mass media and other sources of information? What sources of information do they typically rely on for different types of issues?

3. What abortion-related knowledge, perceptions and practices characterize these women?

## Methods

### Target population and sampling

This was a cross-sectional study with a target population consisting of married women of reproductive age (15-49), residing in four districts in rural eastern India: two in Bihar state (Patna and Saran) and two in Jharkhand state (Lohardaga, and Gumla).^a ^At the time of baseline data collection, Ipas had trained 111 providers in Patna, no providers in Saran, 28 providers in Lohardaga, and 6 providers in Gumla. As a result, safe abortion services were not uniformly available in all study sites. Women using any permanent method of birth control (female sterilization or male sterilization) for more than 36 months prior to the study were excluded from the sample universe.

Research participants were selected from this population using two-stage stratified random sampling. In the first stage, 18 villages from each of the four districts, out of a total of 72 villages, were selected using probability proportional to population size (PPS) sampling. For the second stage, a detailed household listing effort was carried out in each selected village to generate the universe of households with eligible women. Twenty households within each sampled village were subsequently selected using systematic random sampling for a target total of 1440 women.

Prior to study enrollment, informed consent was obtained from all participants. Female research investigators conducted the interviews to increase women's comfort in discussing sensitive issues. To ensure privacy and confidentiality, each respondent was asked to choose a private room or other location where they would be comfortable talking about sensitive topics. If it was not possible to conduct the interview with sufficient privacy, the research team scheduled an appointment with the respondent to return and conduct the interview at a later date. The overall response rate for the study was 98% and did not differ significantly between Bihar (99%) and Jharkhand (97%). This study underwent ethical review and was approved by the Institutional Review Board of the Centre for Media Studies in New Delhi.

### Data collection measures and procedures

Research participants were interviewed during one or two sessions using a pre-tested semi-structured questionnaire. All questions were asked in local languages. The first session focused on collecting quantitative information from all respondents, including socio-demographic and economic characteristics, reproductive history, exposure to mass media, sources of information for various types of issues, and knowledge, attitudes, beliefs, and practices regarding abortion. Women who reported having or attempting an induced abortion during the three years prior to the survey participated in a second interview session. Using a semi-structured interview, information was collected about where women sought abortion counseling and services, abortion methods that were used, and any complications they experienced.

### Data analysis

The four study districts have similar socio-demographic profiles (Table 1), and the primary difference between states is in caste affiliation. Given Bihar and Jharkhand's common history and shared characteristics,^b ^the aggregated data for all four districts across states are shown.

**Table 1:** Percentage of the population with select socio-demographic characteristics in study districts in Bihar and Jharkhand, India

	BIHAR		JHARKHAND	
	**Patna**	**Saran**	**Lohardaga**	**Gumla**

Scheduled Caste*	19.6	12.2	3.5	5.0
Scheduled Tribe*	0.7	0.2	60.3	70.2
Literacy- population^#^	72.5	68.6	68.3	66.9
Literacy- female^#^	63.7	56.9	57.9	57.0
Non-agricultural activity*	20.9	22.3	46.1	41.3

*: Source - Census of India 2001

#: Source - Census of India 2011

Descriptive statistics are reported for both categorical and continuous variables. Categorical variables are analyzed using frequencies, percentages and confidence intervals. For continuous variables, means and standard deviations are reported. Questions about exposure to different types of mass media were asked separately, and as a result, the tables on mass media exposure (Tables 2 and 3) do not sum to 100% since women could have reported exposure to multiple types of media or multiple sources of information. In order to approximate the economic status of the respondents, a standard of living (SLI) index was developed on the basis of ownership of household durables and assets. Households were assigned a score for each asset, and the scores were summed for each household. A high SLI generally means a higher level of income and the ability to acquire other modern amenities that add to one's comfort [36]. Scale scores were computed to describe women's attitudes and beliefs about abortion by summing five-point Likert scales across items and dividing by the number of items. A higher mean score on any scale item indicates a stronger level of agreement with each statement. Cronbach's alpha reliability coefficients were computed for each construct (Table 4), and items that did not support scale reliability were eliminated to improve the reliability score.

**Table 2:** Socio-demographic and reproductive health characteristics of study respondents (N = 1411)

	n (%)	95%CI
Age		
< 25 years	301 (21.3)	19.2 - 23.5
≥ 25 years	1110 (78.7)	76.5 - 80.8
Schooling		
Never Attended	873 (61.9)	59.3 - 64.4
Up to Medium	366 (25.9)	23.7 - 28.2
Secondary & above	172 (12.2)	10.5 - 13.9
Family type		
Nuclear	621 (44.0)	41.4 - 46.6
Joint/Extended	790 (56.0)	53.4 - 58.6
Religion		
Hindu	865 (61.3)	58.8 - 63.9
Muslim	152 (10.8)	9.2 - 12.4
Other	394 (27.9)	25.6 - 30.3
Caste		
General	169 (12.0)	10.3 - 13.7
Scheduled Caste	118 (8.4)	6.9 - 9.8
Scheduled Tribe	447 (31.7)	29.3 - 34.1
Other Backward Class	677 (48.0)	45.4 - 50.1
Worked in past one year		
Yes	319 (22.6)	20.4 - 24.8
No	1092 (77.4)	75.2 - 79.6
Main source of household income		
Own/Shared Farm	641 (45.4)	42.8 - 48.0
Daily Wage	431 (30.6)	28.1 - 32.9
Business	184 (13.0)	11.3 - 14.8
Salaried/pension	139 (9.9)	8.3 - 11.4
No Regular Work	6 (0.4)	0.1 - 0.8
Other	10 (0.7)	0.3 - 1.2
Living standard		
Low	1173 (83.1)	81.2 - 85.1
Medium	168 (11.9)	10.2 - 13.6
High	70 (5.0)	3.8 - 6.1
Mean number of pregnancies (SD)	3.8 (2.1)	--
Mean number of live births (SD)	3.5 (1.9)	--
Experienced spontaneous abortion		
Yes	189 (14.3)	12.4 - 16.2
No	1129 (85.7)	83.8 - 87.6
Experienced induced abortion		
Yes	61 (4.6)	3.5 - 5.6
No	1257 (95.4)	94.2 - 96.5

**Table 3:** Exposure to mass media and other sources of information (N = 1411)

	n (%)	95%CI
Watch Television		
Yes, Regularly	110 (7.8)	6.4 - 9.2
Yes, Sometimes	104 (7.4)	6.0 - 8.7
Never	1197 (84.8)	83.0 - 86.7
Listen to the radio		
Yes, Regularly	117 (8.3)	6.8 - 9.7
Yes, Sometimes	79 (5.6)	4.4 - 6.8
Never	1215 (86.1)	84.3 - 87.9
Read Newspapers		
Yes, Regularly	56 (4.0)	2.9 - 5.0
Yes, Sometimes	35 (2.5)	1.7 - 3.3
Never	1320 (93.5)	92.3 - 94.8
Have women's club/village committee		
Yes	518 (36.7)	34.2 - 39.2
No	863 (61.2)	58.6 - 63.7
Don't know	30 (2.1)	1.4 - 2.9
Attend club/community meetings		
Yes, Regularly	232 (16.4)	14.5 - 18.4
Yes, Sometimes	94 (6.7)	5.4 - 8.0
Never	192 (13.6)	11.8 - 15.4
Go to the hatt (bazar)/market		
Yes, Regularly	486 (34.4)	32.0 - 36.9
Yes, Sometimes	190 (13.5)	12.5 - 15.2
Never	735 (52.1)	49.5 - 54.7

**Table 4:** Sources of information by type of issue (N = 1411)

	State Issues		Local Issues		Family Planning Information		Abortion Information	
	**n (%)**	**95%CI**	**n (%)**	**95%CI**	**n (%)**	**95%CI**	**n (%)**	**95%CI**

Mass media	299 (21.2)	19.1 - 23.3	-	-	256 (18.1)	16.1 - 20.1	61 (4.3)	3.3 - 5.4
Market place	44 (3.1)	2.2 - 4.0	-	-	-	-	-	-
Villagers	511 (36.2)	33.7 - 38.7	463 (32.8)	30.4 - 35.3	-	-	-	-
Neighbors	403 (28.6)	26.2 - 30.9	819 (58.0)	55.5 - 60.6	-	-	-	-
Health provider	-	-	-	-	96 (6.8)	5.5 - 8.1	62 (4.4)	3.3 - 5.5
Community level source/activity^§^	-	-	58 (4.1)	3.1 - 5.2	721 (51.1)	48.5 - 53.7	200 (14.2)	12.3 - 16.0
Family and friends	262 (18.6)	16.5 - 20.6	96 (6.8)	5.5 - 8.1	354 (25.1)	22.8 - 27.4	65 (4.6)	3.5 - 5.7
Don't know	30 (2.1)	1.4 - 2.9	-	-	-	-	-	-
No information	-	-	-	-	711 (50.4)	47.8 - 53.0	1232 (87.3)	85.6 - 89.1

^§^Community-level source/activity includes women's groups, community clubs, NGO workers, community health workers, and posters or billboards

**Figure 1:**
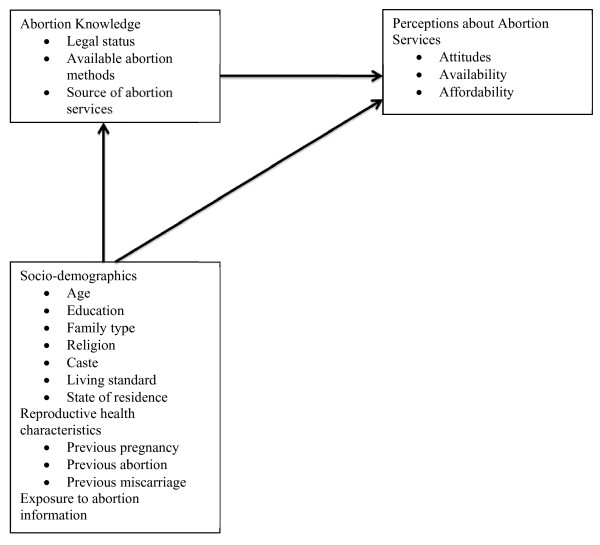
**Conceptual model for factors associated with perceptions about abortion services**.

Multiple linear regression was used to determine the factors associated with perceptions about abortion; standardized Betas and p-values are reported. This study uses three outcome measures of enabling resources considered to be measures of potential access to abortion services: perceived availability of abortion services, perceived affordability of abortion services, and favorable attitudes toward abortion. The conceptual model (Figure 1) shows the hypothesized relationship between knowledge about abortion and perceptions, with socio-demographic and reproductive health characteristics as confounders.

## Results

### Socio-demographic, economic, and reproductive characteristics

Table 2 presents the socio-demographic characteristics of the study participants. Over half of the women in the combined sample were between the ages of 25 and 34 and tended to live either in joint^c ^or extended family households (56%) (Table 2). The majority identified as Hindu (61%) while 11% identified as Muslim, and within the Other category, 22% identified as Sarna^d ^and 6% as Christian (Table 2). More than three-quarters of the sample belonged to either the Other Backward Classes (48%) or to Scheduled Tribes (32%), and 62% of the women never attended school (Table 2). Over 80% of the women fell into the low standard of living category; the main source of household income was from owning a farm (40%) or from a daily wage (31%) (Table 2). Most women reported that they did not work outside their homes in the past year (77%) (Table 2). Women reported a mean of 3.8 pregnancies (SD = 2.1) and 3.5 live births (SD = 1.9) (Table 2). Fourteen percent of women reported at least one spontaneous abortion, while induced abortions were reported by only 5% of women (Table 2).

### Mass media exposure and other sources of information

Women reported very little exposure to mass media (Table 3). Exposure to radio and television was only 14% and 15%, respectively, and newspaper exposure was even lower (6%) (Table 3). Overall, only 23% of women reported that they ever attend a women's club or community meetings (Table 3), but significant differences were seen by state. In Jharkhand, 43% of women reported attending these meetings, but in Bihar, only 3% reported attendance (data not shown). In addition, over half of the women report that they never go to the market (Table 3), and the majority of these women (70%) were from Bihar (data not shown).

### Sources of information for news and reproductive health issues

In addition to gathering data on women's exposure to mass media and other sources of information, we examined how their sources of information differed based on the type of issue (Table 4). For information about their state (e.g., politics, sports), women relied most heavily on villagers (36%), neighbors (29%), mass media (21%) and family and friends (19%) (Table 4). A similar pattern emerged for local issues, with most women relying on their neighbors (58%) or villagers (33%) for information (Table 4). Half of the respondents reported receiving information on family planning during the last year (Table 4). Most of this information was received through community-level sources, including Accredited Social Health Activists (ASHAs) and Anganwadi workers (AWWs) (23%) and Auxiliary Nurse Midwives (ANMs) (21%) (data not shown). Family members and friends were also common sources of information (25%) (Table 4). When asked about having received messages related to abortion, only 13% of women recalled receiving some information on abortion issues during the past year (Table 4). For those who were exposed to messages, the sources of information were similar to the findings on family planning. Specifically, 14% received information from community-level sources, including ANMs (7%), ASHAs and AWWs (5%) (data not shown), and 5% received information from family members and friends (Table 4).

### Knowledge, attitudes, beliefs regarding safe abortion access

Despite the fact that the Indian parliament passed the Medical Termination of Pregnancy Act in 1971, women's knowledge about the legal aspects of abortion is very low (Table 5). Fewer than half of women (41%) knew that abortion is legal. Some women also erroneously believed that abortion is legal only for married women (5%), and only 2% knew that abortion was legal up to 20 weeks of gestation (Table 5). In addition, women were not knowledgeable about abortion methods. While 11% had heard that abortion could be performed by tablets (medical abortion), less than 1% of women were aware of surgical methods of abortion such as dilation and curettage (D&C) or manual vacuum aspiration (MVA) (Table 5). Despite low knowledge about the legality of abortion and specific methods used, almost half of women (46%) were able to correctly name a place where abortion services are available (Table 5).

**Table 5:** Respondent's knowledge about legal aspects, methods and sources of abortion (N = 1411)

	n (%)	95%CI
Legality of Medical Termination of Pregnancy (MTP)		
Yes, legal	505 (35.8)	33.3 - 38.3
Yes, legal if woman is married	66 (4.7)	3.6 - 5.8
No, illegal	676 (47.9)	45.3 - 50.5
Don't know	164 (11.6)	9.9 - 13.3
Gestation limits for legal MTP		
Correct knowledge	27 (1.9)	1.2 - 2.6
Incorrect knowledge	1003 (71.1)	68.7 - 73.5
Don't know	381 (27.0)	24.7 - 29.3
Heard of abortion method		
Surgical method	11 (0.8)	0.3 - 1.2
Medical abortion	151 (10.7)	9.1 - 12.3
Don't know	1249 (68.5)	86.9 - 90.2
Know source of abortion services		
Yes	652 (46.2)	43.6 - 48.8
No	759 (53.8)	51.2 - 56.4

Data on women's attitudes and perceptions about abortion are shown in Table 6. Women scored above the scales' midpoint for three categories, or constructs, hypothesized to be enabling resources for potential access to safe abortion services. The three constructs considered existing enabling resources were: perceived risk associated with unsafe abortion, social support and self-efficacy. Women perceived significant health risks of unsafe abortion (mean = 3.9, SD = 1.03), and many agreed that they have strong family social support systems (mean = 3.6, SD = 0.88). Women also reported a high level of self-efficacy (mean = 3.8, SD = 0.80) in making reproductive decisions, including accessing safe abortion services.

**Table 6:** Respondent's attitudes and perceptions related to access to safe abortion services (N = 1411)

	Cronbach's Alpha	Mean	(SD)
Perceived availability^1 ^of abortion services	0.32	2.5	(0.92)
Perceived affordability^2 ^of abortion services	0.57	2.0	(1.04)
Perceived health risks^3 ^of unsafe abortion	0.56	3.9	(1.03)
Favorable attitude^4 ^towards abortion	0.39	2.1	(1.01)
Perceived self-efficacy^5 ^with respect to FP and abortion	0.66	3.8	(0.80)
Perceived social support^6 ^within family for abortion	0.31	3.6	(0.88)
Perceived social norms^7 ^regarding abortion	0.28	2.3	(0.76)

^1^Availability is the extent to which the service is found in a pre-defined area. Availability of services includes respondents' opinion on: access to abortion services at facilities close to their village, access at urban private clinics compared to rural health centers, and availability of information on abortion providers.

^2^Affordability of abortion services includes respondents' perception of the cost of abortion services compared to other health services.

^3^Perceived health risks includes respondents' understanding of: the severity of postabortion complications due to unsafe abortion, comparative risk to women's health if abortion is done at a late stage of pregnancy, and harmful effects of repeated unsafe abortion.

^4^Attitude towards abortion includes women's perception of terminating unintended pregnancy and experience of feelings of guilt associated with abortion.

^5^Self-efficacy includes respondent's confidence and ability to negotiate with spouse and medical doctors to discuss her own reproductive and contraceptive choices, including number of children, contraceptive methods, birth spacing and abortion issues.

^6^Social support within the family includes women's perception regarding spousal and parental support in accessing abortion, if required.

^7^Social norms are the behavioral standards, which exist in the community for an individual to follow (29). Social norms include perceived community acceptability of abortion.

Conversely, women scored below the midpoint for the remaining four constructs hypothesized to be barriers to accessing safe abortion services. Barriers included individual and societal norms about abortion that were perceived as negative, and the perceived lack of availability and affordability of safe abortion services. While women showed strong self-efficacy and social support, societal norms (mean = 2.3, SD = 0.76) and individual attitudes (mean = 2.1, SD = 1.01) were not seen as favorable towards abortion (Table 6). The perceived availability (mean = 2.5, SD = 0.92) and affordability (mean = 2.0, SD = 1.04) of abortion services were uniformly mentioned as a major concern (Table 6). The majority of women reported that abortion services are primarily available in urban clinics and hospitals, and providers normally charge more money compared to other related services (data not shown). These perceptions signify a lack of enabling resources and act as barriers to access to abortion services.

The bivariate association between women's characteristics and perceptions about abortion availability, affordability and attitudes is presented in Table 7. Exposure to an abortion message is positively associated with attitude and perceived availability scores; however, exposure to an abortion message is negatively associated with perceived affordability (Table 7). Perceived availability of abortion services is also positively associated with knowledge of the legal status of abortion in India (p < 0.05) (Table 7). However, women who can accurately name a source of abortion services have significantly lower perceived availability scores than women who do not know a source of services (2.38 and 2.63, respectively; p < 0.01) (Table 7). In addition to the negative association with exposure to an abortion message, perceived affordability is associated with standard of living. Women who have a medium standard of living perceive abortion services to be less affordable (mean score = 1.84) than women in the low (mean score = 2.02) or high (mean score = 1.97) standard of living groups (p < 0.05) (Table 7). Favorable attitudes toward abortion were associated with socio-demographic characteristics such as higher levels of education, being in the Other religion category, and membership in the General caste group (Table 7). Favorable attitudes were also associated with knowledge of the legal status of abortion, knowledge of a source of abortion services, knowledge of an abortion method, and exposure to an abortion message (Table 7).

**Table 7:** Bivariate associations between socio-demographic and reproductive health characteristics and potential barriers to accessing abortion services

		Perceived availability			Perceived affordability			Favorable attitude		
	**n**	**mean**	**SD**	**t**	**mean**	**SD**	**t**	**mean**	**SD**	**t**

Age										
< 25 years	301	2.52	0.93	--	2.02	1.05	--	2.14	1.04	--
≥ 25 years	1110	2.51	0.92	0.48	2.00	1.04	0.38	2.12	1.00	0.39
Schooling										
Never attended	873	2.51	0.91	-0.30	2.03	1.05	-1.50	2.06	0.97	2.97**
Up to medium	366	2.48	0.92	0.75	1.94	0.99	1.29	2.19	1.04	-1.39
Secondary & above	172	2.55	0.95	-0.56	1.96	1.09	0.49	2.31	1.10	-2.57*
Type of family										
Nuclear	621	2.55	0.88	--	2.06	1.04	--	2.13	1.00	--
Joint/Extended	790	2.48	0.94	1.14	1.95	1.04	1.79	2.13	1.02	0.021
Religion										
Hindu	865	2.50	0.89	0.60	1.98	1.02	0.59	2.07	0.97	0.15*
Muslim	152	2.48	1.02	0.41	2.04	1.16	-0.54	2.07	1.11	-0.69
Other	394	2.55	0.93	-1.04	2.01	1.05	-0.30	2.26	1.05	-3.15**
Caste										
General	169	2.42	0.83	1.41	2.10	1.11	-1.38	2.23	1.06	-2.12*
Scheduled Caste	118	2.49	0.87	0.21	2.05	1.02	-0.61	2.00	1.01	1.39
Scheduled Tribe	447	2.58	0.94	-1.77	1.96	1.06	0.72	2.20	1.04	-1.74
Other Backward Class	677	2.49	0.93	0.61	1.98	1.02	0.56	2.06	0.97	2.23*
Living Standard										
Low	1173	2.52	0.91	-0.51	2.02	1.05	-1.89	2.15	1.00	-1.93
Medium	168	2.49	0.92	0.29	1.84	0.99	2.03*	2.01	1.02	1.55
High	70	2.47	0.89	0.44	1.97	1.03	0.23	2.00	1.01	1.00
State										
Jharkhand	700	2.55	0.95	--	2.02	1.04	--	2.13	1.04	--
Bihar	711	2.47	0.88	1.66	1.98	1.04	0.74	2.11	0.97	0.39
Knowledge on legal status										
No	840	2.47	0.99	--	1.99	1.07	--	1.93	0.93	--
Yes	571	2.58	0.80	-2.09*	2.00	1.00	-0.20	2.43	1.06	-9.26**
Know source of abortion services										
No	759	2.63	0.94	--	2.00	1.07	--	2.06	0.98	--
Yes	652	2.38	0.87	5.08**	1.99	1.00	0.23	2.20	1.04	-2.53*
Ever pregnant										
No	93	2.49	0.89	--	2.20	1.04	--	2.13	0.99	--
Yes	1318	2.52	0.92	-0.29	1.98	1.04	1.91	2.12	1.01	0.08
Ever had abortion										
No	1350	2.51	0.92	--	2.00	1.05	--	2.13	1.01	--
Yes	61	2.55	0.96	-0.38	1.79	0.94	1.56	1.86	0.89	1.00
Ever had miscarriage										
No	1222	2.51	0.91	--	1.99	1.05	--	2.12	1.01	--
Yes	189	2.53	0.92	-0.30	2.02	1.00	-0.35	2.11	0.97	0.12
Know any modern method of abortion										
No	1249	2.48	0.92	--	2.03	1.05	--	2.05	0.98	--
Yes	11	2.88	1.00	-1.42	1.73	1.14	0.94	3.00	1.43	-3.01**
Exposure to message on abortion										
No	1232	2.48	0.93	--	2.04	1.06	--	2.03	0.96	--
Yes	179	2.74	0.75	-3.59**	1.74	0.88	3.52**	2.84	1.05	-9.84**

**: *p *< 0.01; *: *p *< 0.05

Table 8 shows the results of multiple linear regression models of the factors associated with three constructs: perceived availability, perceived affordability and favorable attitude toward abortion. These three constructs were chosen as outcome measures because they are thought to be barriers to accessing safe abortion services. Adjusting for socio-demographic and reproductive health characteristics, women who knew an abortion method were more likely to perceive that abortion services were available compared to those who did not know a method (β = 0.079; p = 0.02) (Table 8). However, women who knew a correct source of abortion services were less likely to perceive that abortion services were available compared to those who did not know a source (β = -0.168; p < 0.01) (Table 8). The perceived affordability model shows different associated factors. Women who are in the Other religion category were more likely to perceive that abortion services were affordable compared to Hindu women (β = 0.119; p = 0.04) (Table 8). In addition, women who are Scheduled Tribe and who have ever been pregnant are significantly less likely to perceive that abortion services were affordable compared to general caste and nulligravidas women, respectively (Table 8). In the attitudes model, women who are in the Other religion category were also more likely to have a favorable attitude toward abortion compared to Hindu women (β = 0.145; p = 0.01) (Table 8). In addition, knowing an abortion method and reporting exposure to an abortion method were positively associated with favorable attitudes toward abortion (Table 8). Predictors of negative attitudes toward abortion included membership in the Other Backward Class caste group compared to women in the general caste group, and women in the medium and high living standard groups compared to the poorest women (Table 8).

**Table 8:** Multiple linear regression results of factors associated with potential barriers to accessing abortion services

	Perceived availability		Perceived affordability		Favorable attitude	
	**Standardized β**	**p-value**	**Standardized β**	**p-value**	**Standardized β**	**p-value**

Constant	--	.000	--	.000	--	.000
Age						
< 25 years (R)						
≥ 25 years	-0.010	0.730	-0.007	0.798	-0.009	0.747
Schooling						
Never attended (R)	--	--	--	--	--	--
Up to medium	-0.024	0.388	-0.029	0.314	0.012	0.652
Secondary & above	-0.004	0.886	-0.010	0.737	0.020	0.498
Type of family						
Nuclear (R)	--	--	--	--	--	--
Joint/Extended	-0.28	0.315	-0.042	0.137	-0.001	0.967
Religion						
Hindu (R)	--	--	--	--	--	--
Muslim	-0.012	0.677	0.001	0.966	0.004	0.881
Other	-0.054	0.344	0.119*	0.038	0.145**	0.009
Caste						
General (R)	--	--	--	--	--	--
Scheduled Caste	0.013	0.699	-0.027	0.426	-0.057	0.080
Scheduled Tribe	0.076	0.289	-0.212**	0.004	-0.120	0.088
Other Backward Class	0.017	0.701	-0.070	0.110	-0.089*	0.035
Living Standard						
Low (R)	--	--	--	--	--	--
Medium	-0.012	0.656	-0.044	0.118	-0.082**	0.002
High	-0.022	0.442	-0.004	0.896	-0.073**	0.007
State						
Jharkhand (R)	--	--	--	--	--	--
Bihar	-0.054	0.172	-0.052	0.190	0.094*	0.014
Knowledge on legal status						
No (R)	--	--	--	--	--	--
Yes	0.065*	0.018	0.026	0.341	0.202**	0.000
Know source of abortion services						
No (R)	--	--	--	--	--	--
Yes	-0.168**	0.000	-0.017	0.544	-0.001	0.969
Know an abortion method						
No (R)	--	--	--	--	--	--
Yes	0.079*	0.015	-0.013	0.684	0.070*	0.023
Expose to abortion message						
No (R)	--	--	--	--	--	--
Yes	0.056	0.087	-0.082	0.014	0.182**	0.000
Ever pregnant						
No (R)	--	--	--	--	--	--
Yes	0.004	0.881	-0.056*	0.048	-0.016	0.562
Ever had abortion						
No (R)	--	--	--	--	--	--
Yes	-0.014	0.595	-0.024	0.381	-0.031	0.235
Ever had miscarriage						
No (R)	--	--	--	--	--	--
Yes	0.001	0.981	0.011	0.680	-0.016	0.537

**: *p *< 0.01; *: *p *< 0.05; R = Reference Category

### Characteristics of past abortion attempts

For the subset of women (n = 61) who reported having or attempting an induced abortion during the three years prior to the survey, a second interview was conducted on abortion practices for their last abortion (Table 9). Overall, 38% of women first attempted to induce their abortion at home (Table 9), and 39% of these women reported that they subsequently experienced post-abortion complications (data not shown). The majority (89%) consulted a doctor or health worker for advice on how to obtain an abortion (Table 9), but only 19% of these providers were posted at facilities that were known to be approved to provide safe abortion services (data not shown). Women learned about these providers through their husbands (61%) and/or from other family members or friends (50%) (Table 9). The type of health service providers women consulted most frequently were doctors in private clinics or nursing homes (59%), and women reported that the final decision to go to the provider was made by both husbands (74%) and themselves (70%) (Table 9). Women typically travelled to another town for services (65%) and underwent either a medical abortion (45%) or a surgical method (45%) (Table 9). The majority of women reported no complications (72%) (Table 9). Of the women who reported complications, 47% sought treatment and 38% required hospitalization (Table 9). Though abortion services are virtually free at government facilities, women reported that the average total cost for an abortion was Rs.1348 (SD = Rs. 851), though the cost varied greatly (Table 9). Major expenses were primarily associated with the procedure itself (mean = Rs. 572), travel costs (mean = Rs. 439), and medicine (mean = Rs. 371) (data not shown).

**Table 9:** Characteristics of respondent's last abortion (N = 61)

	n (%)	95%CI
Attempted abortion at home		
Yes	23 (38)	25.5 - 49.9
No	38 (62)	50.1 - 74.5
Consultation with a doctor/health worker		
Yes	54 (88.5)	80.5 - 96.5
No	7 (11.5)	3.5 - 19.5
How did you learn of this provider?		
Husband	33 (61.1)	48.1 - 74.1
Family members/friends	27 (50.0)	-0.6 - 11.7
Local providers/health workers	8 (14.8)	5.3 - 24.3
Other	4 (7.4)	0.4 - 14.4
Who made the decision to go to the provider?		
Self	38 (70.4)	58.2 - 82.6
Husband	40 (74.1)	62.4 - 85.8
Types of health service providers consulted		
Doctor - District hospital	9 (16.7)	6.7 - 26.6
Doctor - Primary health center	4 (7.4)	0.4 - 14.4
Doctor - Private clinic or nursing home	32 (59.3)	46.2 - 72.4
Nurse/Auxiliary Nurse Midwife (ANM)	4 (7.4)	0.42 - 14.39
Unqualified local provider^§^	5 (9.3)	1.5 - 17.0
Location		
Same village	12 (22.2)	11.1 - 33.3
Other town	35 (64.8)	52.1 - 77.6
Other village	7 (13.0)	4.0 - 21.9
Abortion method		
Surgical method	24 (45.3)	31.9 - 58.7
Medical abortion	24 (45.3)	31.9 - 58.7
Other	6 (11.1)	2.73 - 19.5
Complications		
Yes	17 (27.9)	16.6 - 39.1
No	44 (72.1)	60.9 - 83.4
Treatment sought for complications^1^		
Yes	8 (47.1)	23.3 - 70.8
No	9 (52.9)	29.2 - 76.7
Hospitalization sought for complications^1^		
Yes	3 (37.5)	3.9 - 71.1
No	5 (62.5)	28.9 - 96.1
Average cost (SD)	1348 (851.4)	--

^1^Percent reported among those who indicated complications with their abortion

^§^Includes chemist/pharmacist, rural medical practitioner (RMP), dai/midwife

## Discussion

The goal of this study was to develop an evidence base to understand issues related to the accessibility of safe abortion services in Bihar and Jharkhand from the perspective of rural Indian women. We discuss our findings about women's socio-demographic characteristics, exposure to mass media and abortion-related knowledge, attitudes, beliefs, and practices. Each of these findings has implications for the design of a woman-centered BCC intervention. Limitations of the study are also discussed below.

### Women's socio-demographic, economic, and reproductive characteristics

This study's two-stage stratified random sample provided a snapshot of the socio-demographic and economic characteristics of married women of reproductive age in four districts in Bihar and Jharkhand. These characteristics must be taken into account in planning BCC interventions that aim to increase access to safe abortion services. The overwhelming majority of women are disadvantaged members of the Scheduled Tribes, Scheduled Castes or Other Backward Classes, and their monthly incomes are very low. More than half of the women interviewed had never attended school and were likely to be illiterate or of very low literacy. The implication for any BCC intervention is that printed materials, if they are used at all, need to be designed for very low literacy users (e.g., rely heavily on illustrations, contain only simple text).

Over three-quarters of the women reported that they did not work outside the home, which suggests that BCC interventions will be unable to reach women in work settings. This finding also suggests that many women do not have independent access to income for purchasing reproductive health services. Women's family types included both nuclear and joint family structures, and the women belonged to different religions, including Hinduism, Sarna, and Islam. These differences should be further explored to determine whether BCC interventions should be segmented for audiences based on these characteristics (e.g., a woman living in a joint household might benefit more from an approach that includes educating her in-laws about safe abortion).

### Mass media exposure and other sources of information

Limited exposure to mass media, including television, radio and newspapers suggests that women cannot be effectively reached through an electronic or print media campaign. A better approach may be to reach women in community settings. However, state differences suggest that communities use dissemination channels differently. For example, while more than half of women in Jharkhand attend women's clubs or village committee meetings, community meetings, and/or go to the market, women in Bihar were not as engaged in these activities. This suggests that BCC interventions should make use of these channels where they exist, and investment in the development of these community channels should be considered where they do not already exist.

Important secondary audiences such as AWWs, ANMs, ASHAs as well as family and friends can be influential intermediaries in reaching the primary audience of married women of reproductive age. Since these individuals typically provide antenatal care, childhood immunizations, and family planning services in their communities, it is intuitive that they were the most frequently cited sources of information for sensitive issues such as family planning and abortion. Other potential secondary audiences may include the villagers and neighbors on whom women rely for information on state and local issues. Use of secondary audiences as social influencers may also help address the challenges of reaching a low literacy audience if AWWs and/or ANMs have higher levels of education relative to other women in the village. Secondary audiences may also be a useful approach for influencing the social norms and social support in a community, as they help women make important decisions on health and often act as opinion leaders in the community. Our findings on sources of information are mirrored by similar work in other Indian states [25,35].

### Knowledge, attitudes, beliefs, and practices regarding safe abortion access

Our assessment of women's knowledge, attitudes, beliefs, and practices regarding safe abortion access suggest a need for a BCC intervention. Though the MTP Act has existed for four decades in India, more than half of the women in this study were unaware that abortion is legal in India, and almost none of the women were aware of specific aspects of the law. In addition to knowledge gaps that may serve as barriers to accessing safe abortion services, women's attitudes and beliefs also indicated potential barriers to utilization of safe abortion services. These barriers included pragmatic concerns about the availability and affordability of safe abortion services, negative attitudes about abortion, and concerns about stigmatizing social norms. In contrast, existing enabling resources for potential access to safe abortion services included high perceived risk of unsafe abortion, self-efficacy about family planning and abortion decision-making, and perceived social support for safe abortion, especially from family members. Again, our findings are in line with other research in India [25].

The multivariate results provide a more complete picture of the factors associated with enabling resources for potential access to abortion services. Though the finding that knowing where to access abortion services is associated with being less likely to perceive that services are available may seem counterintuitive, it is likely a reflection of the fact that abortion services are not available in villages, and women must travel great distances to access these services. The finding that women who have specific knowledge about abortion, such as knowing an abortion method, are more likely to perceive that services are available and have positive attitudes toward abortion highlights a promising opportunity for BCC interventions to increase knowledge as an enabling resource for access to abortion services. In addition, the finding that exposure to abortion messages is associated with more positive attitudes about abortion emphasizes the importance of these activities in changing attitudes and improving potential access.

To be maximally effective, BCC interventions should draw on the existing enabling resources and address perceived barriers. For example, interventions should primarily focus on promoting the legal aspects of abortion in India and publicize the availability of safe abortion services and technologies at public health facilities. Barriers related to attitudes and beliefs may be more intractable, but women's self-efficacy, perceptions of unsafe abortion-related health risks, and perceived family support can be invoked to support positive messages about reproductive rights. Interpersonal approaches that engage community leaders and influencers may also counteract negative social norms and stigma regarding abortion.

Findings on current abortion practices, while based on a subsample, also have implications for the design of BCC interventions. While 89% of women reported that they consulted with a doctor or health worker about their most recent abortion, many women first attempted to perform abortions at home, as reported in other studies [37]. Women also rely primarily on private doctors instead of public sector providers, which may increase abortion costs, making safe abortion unaffordable. Women may also be receiving surgical abortions in cases where less invasive procedures, such as medical abortion, would be more appropriate.

### Study limitations

Our findings should be viewed within the context of the study's limitations. Household surveys rely on self-report by the respondents, and reporting and recall bias are possible. Like other demographic and social surveys, the incidence of abortion and knowledge of abortion-related information may be under-reported. The findings of this study are based on four selected districts and cannot be generalized to the whole of Bihar and Jharkhand. However, most of the study's findings on women's knowledge, attitudes, behavior, and practice are in line with other published research in these states.

## Conclusions

This study has shown that despite strong and flexible abortion policies, perceived access to safe abortion services can remain low unless information is communicated and a supportive environment is created. Interventions such as BCC, which improve community knowledge about legal aspects, safe providers and methods of abortion, and that support availability of safe abortion services at rural health facilities are needed to improve potential access to services and reduce maternal mortality and morbidity associated with unsafe abortion in India.

Implementing BCC interventions is challenging in settings such as Bihar and Jharkhand where many women are difficult to reach due to restricted mobility, low levels of literacy and limited exposure to mass media. However, this study has shown that women access information through inter-personal communication with family and community members and through existing community health workers. These individuals are important resources that interventions can target to transfer information to the women who need this information most. In addition, interpersonal approaches that engage community leaders and influencers may counteract negative social norms regarding abortion and associated stigma. Collaborative actions of government, NGOs and private partners should capitalize on this potential power of communities to reduce the impact of unsafe abortion on rural women.

## Endnotes

^a ^This study uses baseline data that were collected as part of a larger effort to create and evaluate the impact of a behavior change communication intervention that will educate rural Indian women about safe abortion. To support this larger evaluation effort, which will employ a rigorous pre-post quasi-experimental research design, two districts were selected for intervention based on the availability of safe abortion services (Patna in the state of Bihar and Lohardaga in the state of Jharkhand). Two comparison districts, Saran in Bihar and Gumla in Jharkhand, were subsequently selected because they had similar socio-demographic characteristics at the population level.

^b ^Until 2000, Bihar and Jharkhand were part of the same state, and they continue to share many similarities including high rates of poverty, illiteracy, and infant and child mortality [38,39]. They also have high fertility rates, high unmet need for family planning [38,39], and high estimated rates of abortion [7]. Furthermore, the status and autonomy of women are lower in Bihar and Jharkhand than the country average [40].

^c ^A joint family household is an extended family household in which all of the male members are blood relatives, and all of the female members marry into the family or are unmarried daughters.

^d ^Sarna is a traditional religion of some Scheduled Tribe groups in Jharkhand, characterized by spirit worship.

## Competing interests

The authors declare that they have no competing interests.

## Authors' contributions

SB led all aspects of the study including design, implementation, analysis and writing. KA contributed to study design, analysis strategies and manuscript development. RB participated in analysis, interpretation and writing. JW participated in study implementation and data analysis. All authors read and approved the final manuscript.

## Pre-publication history

The pre-publication history for this paper can be accessed here:

http://www.biomedcentral.com/1471-2458/12/175/prepub
